# Adsorption-Induced Surface Magnetism

**DOI:** 10.1021/acsnano.5c15791

**Published:** 2026-01-30

**Authors:** Miloš Baljozović, Shiladitya Karmakar, André L. Fernandes Cauduro, Mothuku Shyam Sundar, Marco Lozano, Manish Kumar, Diego Soler-Polo, Andreas K. Schmid, Ashutosh V. Bedekar, Pavel Jelinek, Karl-Heinz Ernst

**Affiliations:** † 28501 Empa, Swiss Federal Laboratories for Materials Science and Technology, Überlandstrasse 129, CH-8600 Dübendorf, Switzerland; ‡ Nanosurf Lab, 86889 Institute of Physics of the Czech Academy of Sciences, Cukrovarnická 10, 162 00 Prague 6, Czech Republic; § National Center for Electron Microscopy, 682790 Molecular Foundry, Lawrence Berkeley National Laboratory, Berkeley, California 94720, United States; ∥ Department of Chemistry, The Maharaja Sayajirao University of Baroda, Vadodara 390 002, India

**Keywords:** surface magnetism, chirality-induced spin selectivity, spin-polarized low-energy electron microscopy (SP-LEEM), Hubbard model, chemisorption

## Abstract

We report the emergence of adsorption-induced magnetism from heterohelicene molecules on a nonmagnetic Cu(100) surface. Spin-polarized low-energy electron microscopy measurements reveal spin-dependent electron reflectivity for enantiopure 7,12,17-trioxa[11]­helicene (TO[11]­H) monolayers, indicating the formation of a spin-polarized state localized in the topmost copper layer. Control experiments on clean Cu(100) and TO[11]H on highly oriented pyrolytic graphite show no such effect, excluding artifacts and chirality-induced spin selectivity as origins. Spin-polarized density functional theory calculations with hybrid functionals attribute the magnetism to strong chemisorption, which induces hybridization between the molecular HOMO and copper s- and d-states, driving asymmetric spin-polarized charge redistribution at the interface. An extended Newns–Anderson–Grimley model incorporating on-site Coulomb repulsion in Cu d-orbitals reproduces the emergence of interfacial spin polarization above a threshold interaction strength, highlighting the key roles of hybridization parameters and Coulomb correlation. These findings reveal a mechanism for inducing magnetism at molecule–metal interfaces without inherently magnetic components, offering avenues for engineering spin-polarized states in organic–inorganic hybrid systems.

## Graphical abstract

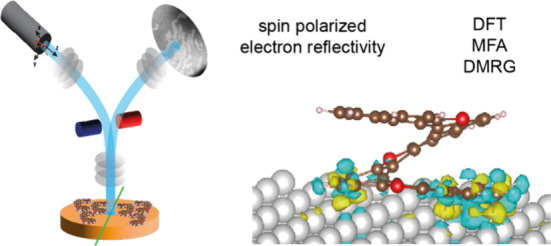

## Introduction

In transition metals, paramagnetism originates from unpaired electrons in partially filled outer d-orbitals. These d-electrons are not only responsible for generating magnetic moments via their intrinsic spin and orbital angular momentum, but they also play a central role in the chemical bonding that governs the structure and properties of solid-state materials.^1^ The relationship between covalent bonding and magnetism was first systematically explored by Linus Pauling, who laid the theoretical foundation for understanding magnetic exchange interactions through chemical bonding concepts.^2^ Since then, both experimental and theoretical advances have significantly deepened our understanding of how electronic structure and bonding contribute to magnetic behavior in complex materials.^3–6^

A major challenge in molecular spintronics is the controlled manipulation of magnetism at the atomic and molecular scales—a requirement for next-generation spin-based logic and quantum technologies. One promising route relies on adsorption-induced magnetism, where nonmagnetic systems acquire magnetic characteristics through charge transfer, orbital hybridization, or symmetry breaking at interfaces. Such effects have been observed for molecules on transition-metal surfaces, including the emergence of spin-polarized states at the Cu/C_60_ interface.^7^

A growing body of work demonstrates that organic molecules can profoundly modify the magnetic properties of metallic substrates. π-Conjugated molecules can enhance exchange interactions and stabilize molecular-mediated magnetic units,^8^ while p_*z*_–d hybridization can even invert interfacial spin polarization and enable spin-selective electron injection.^9^ The adsorption strength further dictates whether molecules harden or soften magnetic coupling, reflecting an interplay between local geometry and metal–molecule hybrid states.^10^ Experiments on Cu-supported ferromagnets additionally show that organic acceptors can reverse magnetization and induce spin polarization via charge-transfer-driven π–d interactions.^11^

Complementary studies on self-assembled monolayers reveal that ordered organic interfaces on noble metals can exhibit significant magnetization,^12,13^ high anisotropy, and spin-selective transport,^14^ emphasizing the generality of molecularly driven magnetic phenomena. Together, these findings underscore the remarkable capacity of molecular adsorption to tailor the interfacial magnetism in metallic systems. Despite this progress, the fundamental mechanisms governing molecular-induced magnetism in copper remain insufficiently understood, motivating a detailed investigation of how specific molecular interactions generate and control magnetic states on Cu surfaces.

Chiral organic molecules, such as helicenes, offer distinct advantages in the context of spin-dependent phenomena due to their intrinsic handedness and extended π-conjugated frameworks.^15,16^ These structural features enable efficient electronic coupling with metal substrates and facilitate spin-selective interactions via the chirality-induced spin selectivity (CISS) effect.^17,18^ While helicenes have been extensively studied for their optical and electronic properties,^19,20^ their potential to induce or host magnetic moments upon adsorption on metal surfaces remains largely unexplored—despite detailed investigations into their adsorption behavior and self-assembly on various substrates.^21,22^

Recent work by Safari et al. demonstrated that heptahelicene exhibits partial enantioselective adsorption on Co islands supported on Cu(111), depending on the direction of out-of-plane magnetization.^23^ Although the CISS effect is typically associated with spin-selective electron transport,^24–28^ magneto-chiral selectivity during adsorption processes may also fall under this umbrella.^29^ Together, such studies highlight the potential of hybrid organic–metal systems to exhibit emergent magnetic behavior, driven by interfacial charge redistribution and the ordered arrangement of chiral molecules at the surface.

Various techniques are available for studying surface magnetism, among which spin-polarized low-energy electron microscopy (SP-LEEM) is particularly well-suited for surface-sensitive investigations.^30,31^ Owing to the short inelastic mean free path of ballistic electrons in solids,^32^ SP-LEEM is highly surface sensitive, with the top few atomic layers of the sample primarily determining image contrast. In this technique, manipulation of the electron spin state is achieved by directing longitudinally spin-polarized electrons through an electromagnetic deflection system followed by an electron-optical element. Together, these components allow precise orientation of the electron spin in any spatial direction (Figure 1a).

**Figure 1:**
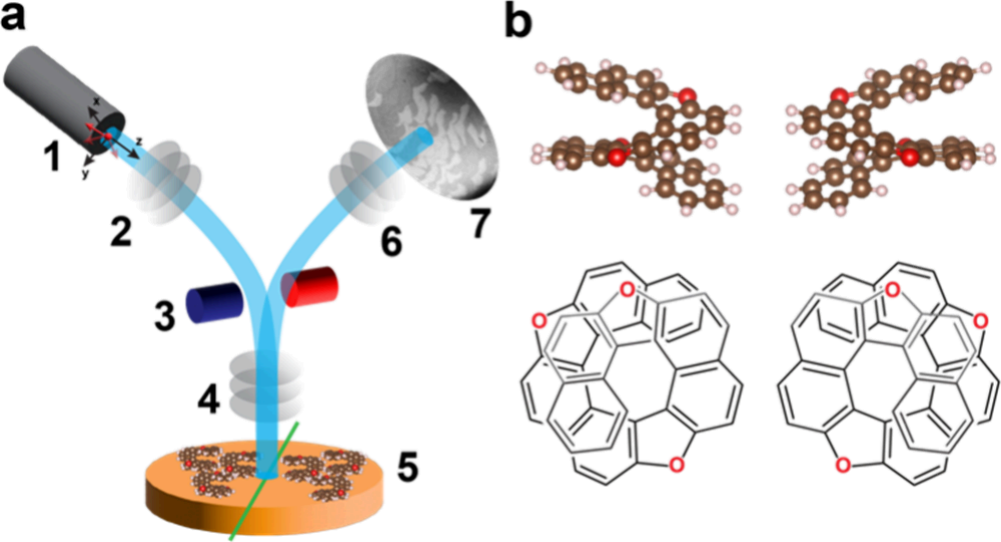
(a) Sketch of SP-LEEM setup, consisting of a spin-polarized electron gun with spin rotator (1), illumination optics (2), beam splitter (3), objective optics (4), the sample (5), imaging optics (6), and a detector/LEEM image (7). (b) Structure of 7,12,17-trioxa[11]­helicene (TO[11]­H), shown as side views of ball-and-stick models (top) and skeletal formulas (bottom) for both enantiomers.

In this work, we report the emergence of adsorption-induced magnetism from heterohelicene molecules (Figure 1b) deposited on a Cu(100) surface. SP-LEEM reveals that adsorption induces a spin-polarized state localized in the topmost atomic layer of copper. Density functional theory (DFT) calculations attribute this magnetism to strong chemisorption, which drives a complex charge back-donation mechanism at the interface, taking place between delocalized metallic s-bands and an antibonding state formed by interaction between the d-band and the HOMO orbital. Importantly, neither the molecular chirality nor direct charge transfer between the metal and adsorbate contributes to the effect.

The origin of the magnetism is rationalized by employing an extended Newns–Anderson–Grimley model, which incorporates electron–electron interaction in spatially localized d-orbitals of the copper surface. The model reveals that strong hybridization of the molecular frontier HOMO orbital with surface states causes a complex charge back-donation between s- and d-bands, which—together with Coulombic repulsion in the localized d-orbitals—gives rise to spin polarization at the interface.

## Results and Discussion

### SP-LEEM Studies

7,12,17-Trioxa­[11]­helicene (TO[11]­H) molecules were adsorbed in their enantiopure forms—either (*P*)-TO­[11]H or (*M*)-TO­[11]­H—onto the Cu(100) surface at room temperature. The formation of an ordered monolayer structure, previously observed by scanning tunneling microscopy (STM),^33,34^ was monitored by low-energy electron microscopy (LEEM).^35^

Figure 2 displays electron reflectivity curves measured for a film of (*M*)-TO­[11]H with slightly more than one close-packed monolayer. When the incident electron energy is below the sample’s work function, all electrons are reflected. Upon reaching the work function threshold, the reflectivity drops sharply, allowing precise determination of the work function from the reflectivity onset. Adsorption of around 1.3 ML of (*M*)-TO­[11]­H reduces the work function by approximately 1.0 eV (Figure 2a). A spatially resolved map of the work function difference between the first and second molecular layers shows a decrease in the work function of about 30 meV on the second layer (Figure S1).

**Figure 2:**
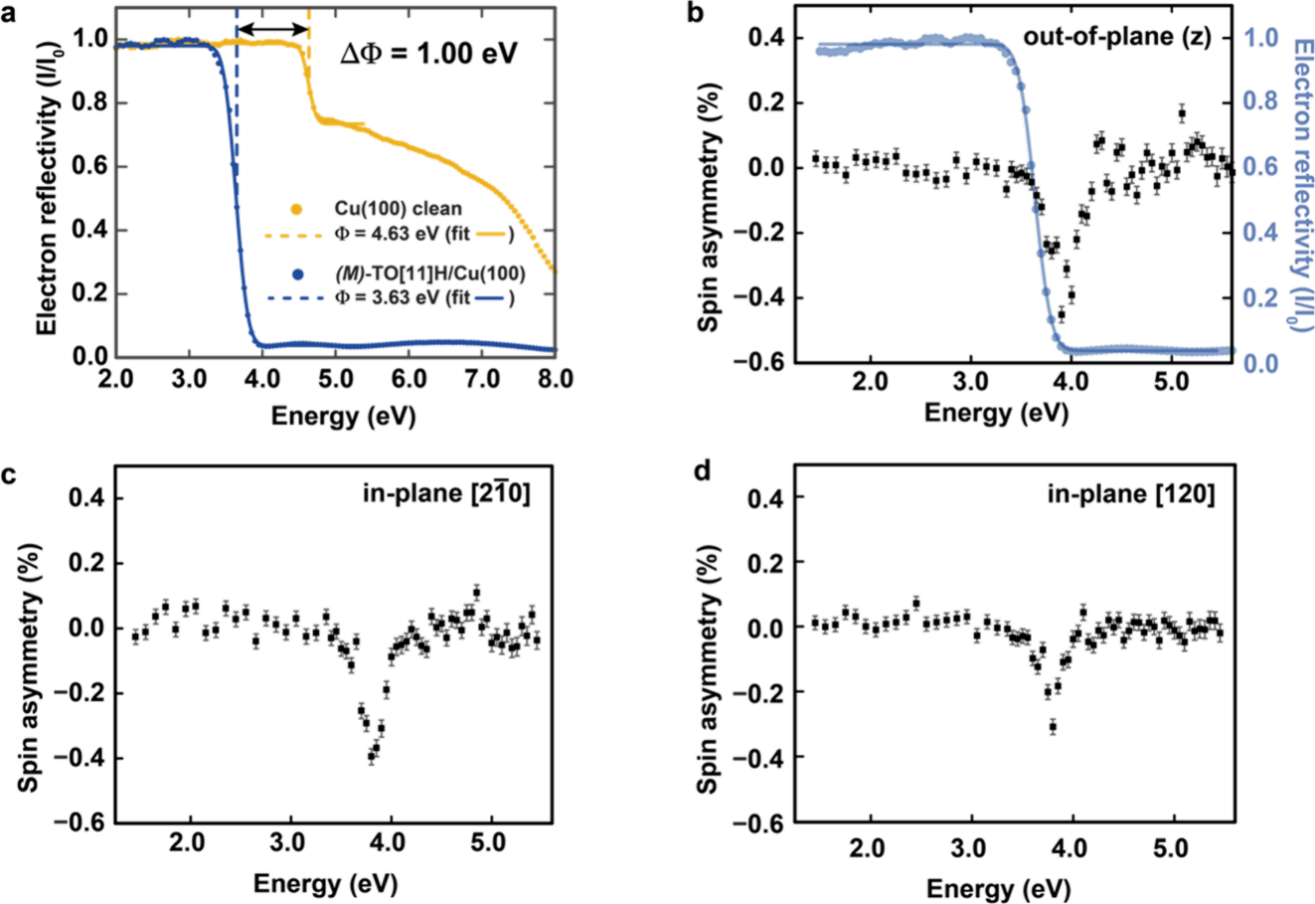
SP-LEEM-measured spin asymmetries. (a) Electron reflectivity curves for clean Cu(100) (yellow) and of a 1.3 ML (*M*)-TO­[11]­H sample (blue). (b) Points of spin asymmetry measurements and intensity of electron reflectivity (blue) with electron impact energy for out-of-plane spin alignment. (c, d) Points of spin asymmetry measurements and intensity of electron reflectivity (blue) with electron impact energy for in-plane spin alignments along [2 $\overset{-}{1}$ 0] and [120] directions.

Point-by-point measurements of spin asymmetry in electron reflectivity with respect to the kinetic energy of the incoming electrons were performed across nearly the entire field of view (Figure S2). The superposition with the reflectivity curve shows that spin asymmetry occurs when electrons are reaching the interface (Figure 2b). Such spin-dependent reflectivity is a hallmark of a ferromagnetic surface, where the electronic structure and density of states differ for spin-up and spin-down electrons near the Fermi level. As in a ferromagnet, the exchange interaction causes energy splitting between majority and minority spin bands; electrons with spins aligned with the majority direction penetrate into the crystal, while those aligned with the minority direction are more likely to be reflected.^36^ Because transmitted electrons are not detected in reflectivity measurements, higher reflectivity corresponds to a lower available density of states. As a result, spin-up electrons are slightly more reflected than spin-down electrons, indicating a spin-selective interaction with the magnetic surface. Moreover, switching the electron polarization sequence leads, as expected, to the opposite effect, resulting in positive values of spin asymmetries near the interface (Figure S3).

Because the number of elastically backscattered electrons depends not only on the surface structure but also on the relative orientation between the spin polarization of the incident beam and the surface magnetization, SP-LEEM allows probing of both in-plane and out-of-plane magnetization components by tuning the spin direction of the incoming electrons. Figure 2c,d displays spin-asymmetry curves for two orthogonal in-plane spin orientations. Both curves show asymmetries comparable in magnitude to the out-of-plane case shown in Figure 2b. Although the observed spin asymmetries are relatively small, they are nonetheless significant. To verify that these signals are not artifacts or background effects, we compared them to the spin asymmetry measurement of reference systems where no magnetism is expected. As shown in Figure 3, clean Cu(100) and TO[11]H molecules adsorbed on highly oriented pyrolytic graphite (HOPG) exhibit no measurable spin asymmetry within the experimental error.

**Figure 3:**
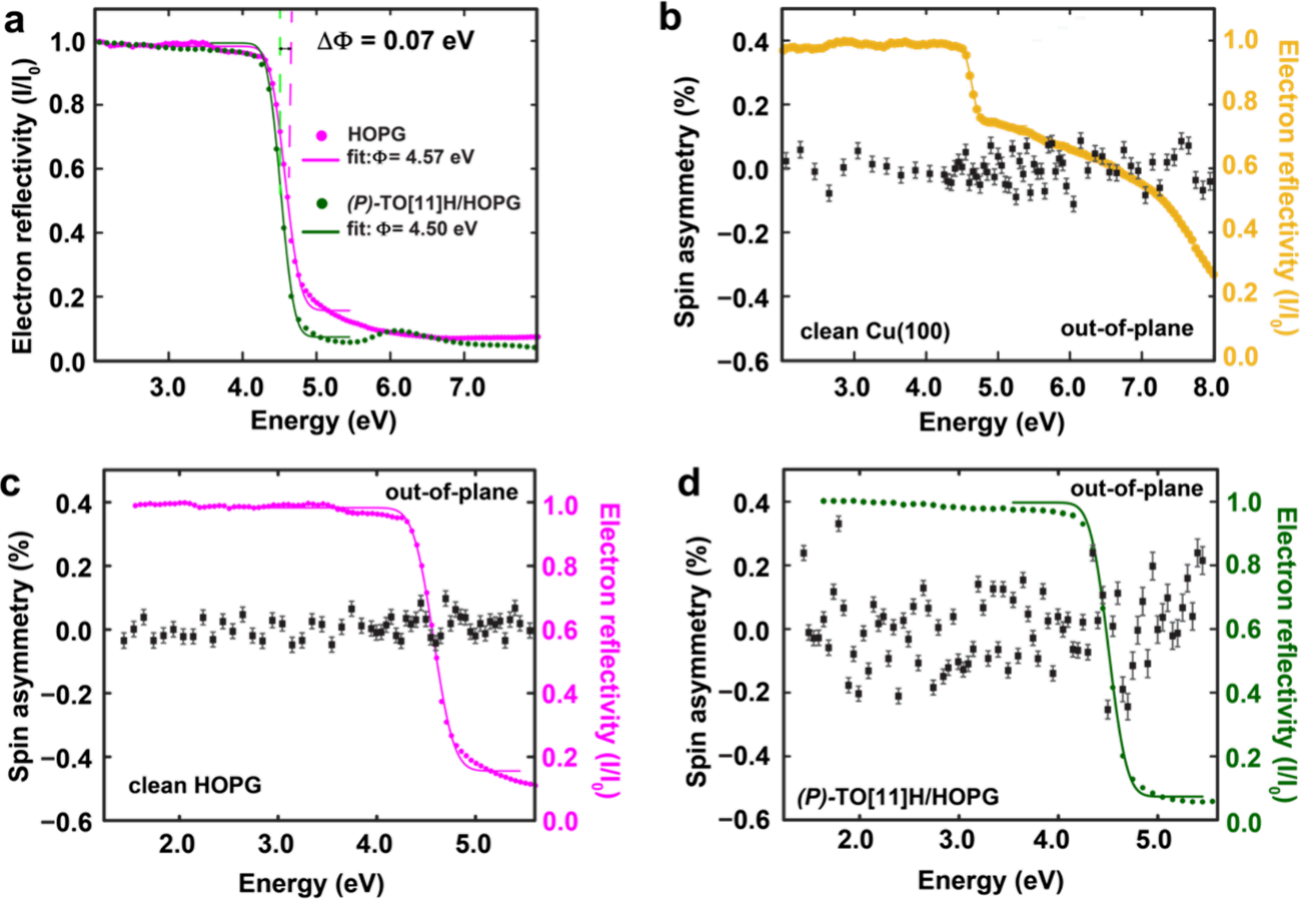
SP-LEEM-measured spin asymmetries for clean surfaces and (*P*)-TO­[11]H on a HOPG. (a) Change of work function of HOPG due to adsorption of (*P*)-TO­[11]H (1 ML). (b–d) Spin asymmetry measurements on Cu(100), HOPG, and a monolayer of (*P*)-TO­[11]H on HOPG, respectively. For all three samples, no asymmetries were observed.

Since the observed effect is identical in sign for both enantiomers (Figure S4), a contribution from the CISS effect to electron reflectivity can be reliably excluded. Previous SP-LEEM studies on heptahelicene adsorbed on Cu(100) also reported no evidence of CISS,^35^ suggesting that the origin of the effect may be more closely linked to topological modifications in the metallic substrate induced by the chiral molecules, rather than arising from the molecular states alone. It is worth noting, however, that spin-resolved photoelectron spectroscopy studies conducted in vacuum on similar systems have produced contradictory results, highlighting the complexity and sensitivity of CISS-related phenomena to experimental conditions.^37,38^ Similar conclusions have been made in a recent study based on break junction experiments and theoretical considerations.^39^

### Results of DFT Calculation

From the optimized model structure, the formation of a bond between Cu atoms and four C atoms of the proximal C6 ring of the TO[11]H molecule is observed. As the bond lengths are only between 2.163 and 2.251 Å, a strong chemisorption of the TO[11]H molecule on the Cu(100) surface is concluded. The binding energy of the model system is calculated to be −4.33 eV, revealing very strong binding of the TO[11]H molecule on the Cu(100) surface. This value is similar to the reported binding of curved, bowl-shaped polycyclic aromatic hydrocarbons (so-called buckybowls) on copper.^40,41^ The calculated charge density difference (Δρ) provides further evidence of strong chemisorption of the TO[11]H molecule on the Cu(100) surface. As shown in Figure S5, significant charge accumulation is observed around the carbon atoms of the six-membered ring in the TO[11]H molecule, accompanied by charge depletion around the underlying Cu atoms. Consequently, this further confirms the robust chemisorption.

The spin-polarized DFT calculations with the Perdew–Burke–Ernzerhof (PBE) functional result in a nonmagnetic ground state with symmetric spin density of states. This suggests that the PBE, which tends to underestimate electronic correlation, is insufficient to capture the magnetic instability at the interface. To more accurately describe the strongly correlated electronic interactions necessary for magnetism, we employed the hybrid PBE0 functional, which incorporates a portion of an exact Hartree–Fock exchange. On a reduced model system (Figure 4a), the PBE0 functional provides a spin-polarized ground state. The computed spin density (isosurface value = 0.02 e/Å^3^, Figure 4b) is primarily localized on the carbon atoms of the chemisorbed ring, with a weaker but non-negligible contribution on the directly bonded Cu atoms beneath.

**Figure 4:**
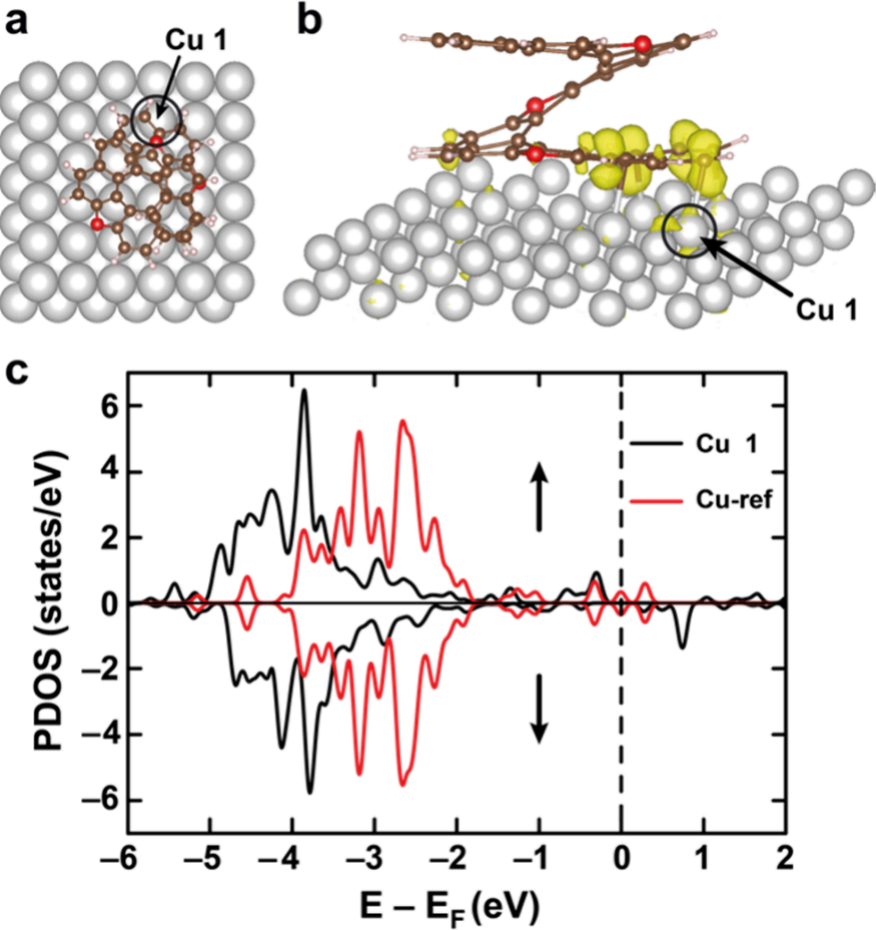
(a) Ball-and-stick representation (top view) of the model system. Gray: Cu, brown: C, red: O, pink: H. (b) Side view of the model system along with the plot of spin density accumulation at the interface (isosurface maximum value set at 0.02 e/Å^3^). (c) Density of states projected to Cu1 attached to TO[11]H and to a Cu atom (Cu-ref) on a clean Cu(100) surface (Cu1 marked by arrows and circles in **a** and **b**).

The absence of spin polarization in PBE, in contrast with its presence in PBE0, suggests that the inclusion of exact exchange in PBE0 is essential for stabilizing the spin-polarized state. To systematically assess the role of exact exchange, the contribution of nonlocal Hartree–Fock exchange (ε_xc_) was varied from 5% to 35%. As shown in Figure S6, no spin density appears at 5% ε_xc_, but it begins to emerge around 15% and grows progressively stronger with increasing ε_xc_. Note that the standard PBE0 functional corresponds to ε_xc_ = 25%.

Figure 4c presents the projected density of states (PDOS) for two copper environments: Cu1, which is directly bonded to the carbon atoms of the TO[11]H molecule’s six-membered ring, and a reference Cu atom from a clean Cu(100) surface. Near the Fermi level, the PDOS is dominated by s-orbital contributions, while the d-orbitals are centered around −3.8 eV for Cu1 and −2.5 eV for the reference Cu atom. The low-energy position of the Cu1 d-band reflects the strong bonding interaction with the TO[11]H molecule, whereas the reference Cu atom presents an electronic structure characteristic of the bare surface.

Moreover, the reference Cu atom exhibits a symmetric, nonspin-polarized PDOS. In contrast, Cu1 displays a pronounced asymmetry in the PDOS below −4 eV, corresponding to its d-orbital region. Note that the apparent suppression of the Cu1 DOS at the Fermi level does not indicate the insulating behavior. It arises from the finite slab thickness (only two layers) and the use of Γ-point sampling, which was necessary because hybrid exchange-correlation functional calculations for metallic slabs are computationally very demanding.

While DFT simulations with hybrid functionals clearly demonstrate that magnetism originates from the hybridization of d-electrons with the HOMO orbital and from enhanced electron–electron interactions in localized states, they do not readily reveal the exact mechanism responsible for interfacial magnetism. To address this limitation, we analyzed model Hamiltonians to gain deeper insight into the magnetic behavior at the molecule/metal interface.

### Results of Model Hamiltonian Calculation

One of the standard models to describe the mechanism of an adsorbate bonding on a metal surface is the Newns–Anderson–Grimley model,^42,43^ which provides insights about the hybridization between the adsorbate molecule and the electronic band structure of the metal surface. According to this model, hybridization with broad metal valence bands, such as s-bands, leads to a broadened adsorbate state. In contrast, interaction with narrow valence bands, such as d-bands, can result in the splitting of the adsorbate state into localized bonding and antibonding states.

To gain deeper insight into the origin of interface magnetization, the Newns–Anderson–Grimley model was extended by incorporating on-site Coulomb electron–electron interactions in both the HOMO (*U*_HOMO_) and the localized d-band (*U*_d_). A detailed description of the model is provided in the Methods section and illustrated in Figure 5a. The results demonstrate that the magnitude of the Coulomb on-site interaction in the d-orbital plays a key role in the emergence of spin polarization at the interface.

**Figure 5:**
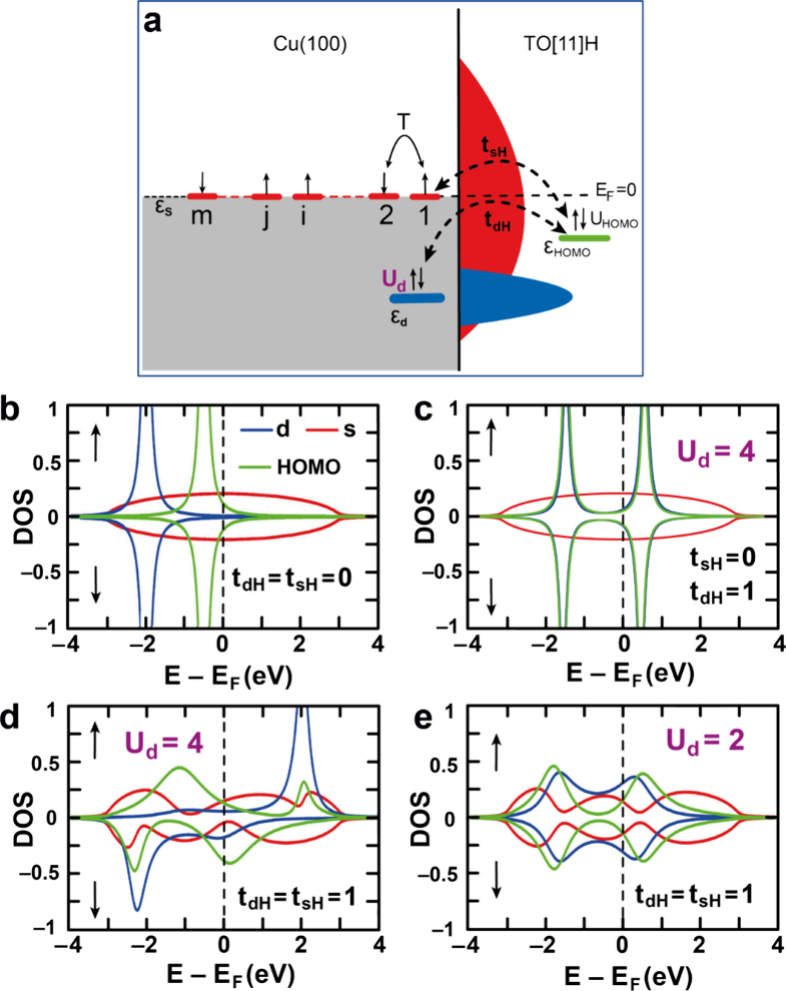
(a) Schematic representation of the model Hamiltonian calculation. The Fermi level, *E*_F_ = 0, is marked with a black dashed line, with the gray-shaded region below *E*_F_ showing occupied substrate bands. Half-filled Cu-s chain (red lines), fully occupied Cu-d (blue), and TO[11]­H-HOMO (green) are shown, with shaded red and blue indicating delocalized s and localized d states. Spin-up/spin-down electrons are shown by the up/down arrows. s-chain hopping T and coupling interactions (*t*_sH_, *t*_dH_) are shown by solid and dashed double-headed arrows, respectively. Projected DOS onto TO[11]­H-HOMO, Cu-d, and frontier Cu-s sites shown for: (b) *U*_d_ = 0, *t*_sH_ = *t*_dH_ = 0; (c) *U*_d_ = 4 eV, *t*_sh_ = 0, *t*_dh_ = 1 eV; (d) *U*_d_ = 4 eV; (e) *U*_d_ = 2 eV, *t*_sh_ = *t*_dh_ = 1 eV. Model parameters used: ε_s_ = 0 eV, *m* = 100, ε_d_ = −2 eV, ε_HOMO_ = −0.5 eV, *T* = 1.5 eV, *U*_d_ = 0 to 4 eV (as indicated), *U*_HOMO_ = 0.

The model parameters were chosen such that they reproduce the qualitative electronic structure of the Cu surface and the molecule, rather than to fit experimental data. To mimic the electronic structure of the Cu(100) surface, delocalized Cu-s states were represented by a broad band centered at the Fermi level with on-site energy of the s-orbitals to ε_s_ = 0 eV and hopping parameter *T* = 1.5 eV to reflect the broad Cu-s bandwidth. One electron per ε_s_-site was assumed to create a half-occupied metallic s-band. Additionally, a doubly occupied Cu-d band was approximated by a localized d-state located at ε_d_ ≈ −2 eV below the Fermi level. The on-site interaction *U*_d_ was scanned from 0 to 4 eV, consistent with reported values for Cu-3d states.^44^ For simplicity, hopping between d-orbitals was neglected to approximate the narrow-band limit.

According to DFT calculations, the molecular HOMO remains doubly occupied and appears at ε_HOMO_ = −0.5 eV. The model Hamiltonian was solved using the self-consistent one-electron mean-field approximation (see Methods). The validity of the one-electron mean-field solution was confirmed by density matrix renormalization group (DMRG) calculations solving the model Hamiltonian.

Figure 5b shows the reference PDOS projected onto the HOMO, frontier s-site, and d-site in the absence of molecule–surface coupling (*t*_sh_ = *t*_dh_ = 0 eV) and Coulomb interactions (*U*_d_ = 0 eV). The singly occupied s-band appears broadened around the Fermi level, while the doubly occupied d-site and HOMO remain localized near −2 and −0.5 eV, respectively. Introducing the molecule–surface interaction via *t*_sH_ and *t*_dH_ leads to hybridization of the HOMO with both the s-band and the d-state.

In the absence of the coupling between HOMO and s-band, *t*_sH_ = 0, hybridization between the HOMO and the d-state (*t*_dh_ = 1 eV) produces bonding and antibonding states, both of which are initially doubly occupied. The magnitude of the on-site Coulomb interaction of the d state, *U*_d_, sets the position of the antibonding state. Figure 5c shows the calculated DOS values for *U*_d_ = 4 eV and *t*_sH_ = 0, where the antibonding state is located above the Fermi level set by the center of the s-band. This results in the net charge transfer from the antibonding state toward the s-band in the mean-field solution. Consequently, the antibonding state becomes completely empty, and two electrons are uniformly distributed into the s-band. Nevertheless, the net charge transfer does not cause magnetization at the interface, as demonstrated by the symmetric DOS in both spin channels shown in Figure 5c.

Interestingly, turning on the coupling between HOMO and the s-band, *t*_sH_ = 1 eV_,_ causes the interface magnetism manifested by strongly asymmetric DOS between spin-up and spin-down channels, as shown in Figure 5d. A strong modification of both bonding and antibonding states originally formed by localized d- and HOMO states is observed. Namely, a strong split of the d-state in spin channels occurs, where the d-state in the spin-up channel is localized at ∼2 eV above the Fermi level, therefore becoming completely empty. On the contrary, the d-state in the spin-down channel is located ∼2 eV below the Fermi level occupied by one electron. Similarly, the HOMO state also becomes asymmetric in both spin channels. The resulting occupations are *n*_d,↑_ = 0.12, *n*_d,↓_ = 0.89; *n*_HOMO,↑_ = 0.76, *n*_HOMO,↓_ = 0.55; and *n*_s,↑_ = 0.46, *n*_s,↓_ = 0.51. These values clearly reflect the spin imbalance, with the d-orbital showing a strong preference for spin-down electrons while the HOMO favors spin-up electrons. These results point out that the coupling between the delocalized s-band and HOMO orbital causes asymmetric charge back-donation in two spin channels, facilitating the emergence of the interface magnetization.

However, the emergence of the interface magnetization also strongly depends on the on-site Coulomb interaction at the d orbital. Figure 5e displays the calculated DOS for the case where the on-site Coulomb interaction at the d-state is set to *U*_d_ = 2 eV. Notably, spin-up and spin-down occupations remain equal, resulting in no spin polarization, as seen from the symmetric DOS of the hybridized states in Figure 5e. According to the simulation, the HOMO loses more charge than the d orbital, and the excess charge in the frontier s orbital is redistributed uniformly across the remaining s orbitals in the tight-binding chain.

It is instructive to analyze how different parameters of the model Hamiltonian affect the emergence of the interfacial magnetism. According to the model, the parameters directly influencing spin polarization in the molecule are (1) hopping parameters *t*_sH_ and *t*_dH_, which determine the degree of orbital hybridization between the molecule and the metal. (2) The on-site Coulomb interaction at the d-orbital of Cu, i.e., *U*_d_. For fixed hopping parameters *t*_sH_ and *t*_dH_, spin polarization is induced at HOMO once *U*_d_ exceeds a certain threshold (*U*_d‑threshold_). Figure S7 provides phase diagrams displaying the magnetic and nonmagnetic regions in the (*U*_d_, *t*_dH_) and (*U*_d_, *t*_sH_) parameter spaces.

The onsite energies ε_s_ and ε_d_ are fixed for a given metal surface, while the hopping parameter between the metal’s s-orbitals depends on the bandwidth of the metal. In contrast, the values of ε_HOMO_ and *U*_HOMO_ are molecule-specific. Our model allows us to explore the conditions under which spin polarization emerges by systematically varying ε_HOMO_ and *U*_HOMO_. To visualize the influence of these parameters, a phase space was constructed involving ε_HOMO_, *U*_HOMO_, and *U*_d_, illustrating how a magnetic moment can develop at the HOMO site, as shown in Figure S8. For simplicity, the hopping parameters *t*_sH_ and *t*_dH_ were fixed at 1. The phase diagram in Figure S8 illustrates the nonmagnetic and magnetic regions, with magnetization strength determined by *M*_HOMO_. While the *U*_HOMO_–*U*_d_ diagram shows a sharp magnetic transition, the ε_HOMO_–*U*_d_ diagram displays a more gradual boundary. The emergence of magnetism at the interface is also confirmed by many-body DMRG calculations resolving the model Hamiltonian for different parameters of *U*_HOMO_ and *U*_d_ (see Figure S9).

In contrast to the modified Stoner mechanism that has been proposed for chemisorbed systems such as Cu/C_60_,^7^ the spin polarization in the present model is primarily governed by three factors: (i) the HOMO–Cu hybridization terms (*t*_sH_, *t*_dH_, Figure S7), (ii) the on-site interaction on Cu-d (*U*_d_, Figure S7), and (iii) the alignment of the HOMO level (ε_HOMO_) relative to the Fermi energy (Figure S8b). Because magnetism arises from the interplay of these parameters, a simple rule of thumb is not readily available. This behavior is more naturally captured within an Anderson-type framework, where the key control parameter is the ratio *U*/*t*. Consequently, the origin of interfacial magnetism extends beyond the single-band Stoner picture based on the product of density of states at the Fermi level and Coulomb interaction, *D*(*E*_F_)*U*.

Nonetheless, our analysis reveals clear qualitative trends. The phase diagrams in Figures S7 and S8 delineate magnetic and nonmagnetic regions and show a threshold value of *U*_d_ ≈ 2 eV for the onset of magnetism. We further find that this threshold depends only weakly on *U*_HOMO_ and on *t*_sH_. In contrast, Figure S7a shows that large *t*_dH_ suppresses the magnetic solution. The magnetic phase boundary in the *t*_dH_–*U*_d_ plane is essentially linear, with a slope of ∼0.3, indicating that magnetism appears only when *U*_d_/*t*_dH_ ≈ 3.33. Increasing *t*_dH_ delocalizes HOMO and consequently shifts the onset of magnetism to larger *U*_d_, reflecting the interplay between hybridization and Coulomb correlation. Finally, the HOMO level must lie close to the Fermi energy but not more than ∼1.5 eV below it.

## Conclusions

Combined SP-LEEM experiments, spin-polarized DFT calculations, and extended Newns–Anderson–Grimley modeling demonstrate that strong chemisorption of heterohelicene molecules on Cu(100) can induce interfacial spin polarization in an otherwise nonmagnetic substrate. The effect arises from hybridization between the molecular HOMO and copper s- and d-states, with Coulomb interactions in the localized d-orbitals driving a spin-symmetry break above a critical threshold. Notably, the magnetism is independent of molecular chirality and does not require direct charge transfer from the adsorbate to the substrate. These findings establish adsorption-induced magnetism as a robust route for engineering spin-polarized states in organic–metal interfaces, expanding the design space for molecular spintronic architectures that do not rely on intrinsically magnetic components.

## Methods

### Experimental Section

TO­[11]H has been synthesized as reported previously.^45^ Enantiomer separation via high-performance liquid chromatography, their circular dichroism, and fluorescence is provided in the Supporting Information (Figures S10–S12). The Cu(100) surface has been cleaned by repetitive argon ion sputtering and annealing at 830 K. In the first several cycles of sputtering, a small amount of oxygen was added for carbon removal. The cleanliness of the substrate was confirmed by Auger spectroscopy. (*M*)- and (*P*)-TO­[11]H molecules were deposited on substrates kept at room temperature from homemade effusion cells held at 480 K. Deposition was performed directly in the SP-LEEM chamber, and coverage was monitored in situ by LEEM intensity/reflectivity changes.

The SP-LEEM measurements were performed at the National Center for Electron Microscopy of Lawrence Berkeley National Laboratory. A cesiated GaAs cathode with the peak of energy distribution of emitted electrons at around *E*_C_^0^ = 1.4 eV was used as an electron source. All samples were prepared under ultrahigh vacuum (UHV) conditions directly in the SP-LEEM chamber with a base pressure lower than 2.0 × 10^–10^ mbar. Samples were initially corrected for the tilt and the electron beam aligned for homogeneous irradiation over the complete field of view with out-of-plane polarized electron spins. In order to avoid beam damage, the measurements were continuously performed on different areas by tediously moving the electron beam laterally over the sample. At each energy, the detector gain was adjusted to compensate for the reflected beam intensity drop due to the reflectivity change. The spin asymmetry of the probed area is defined via the Sherman function as

$$A(\%)=\frac{R_{↑}-R_{↓}}{R_{↓}+R_{↑}}\times 100$$ 1

with *R*_↑_ and *R*_↓_ being the reflectivity of spin up and down electrons, and P the polarization of the beam. Error bars in the plots represent the Standard Error of the Mean obtained from the repeated measurements at the same beam energy.

### Computational Details

#### DFT Calculations

To theoretically investigate the TO[11]­H/Cu(100) interface, we constructed a model system to simulate the interaction between the TO[11]H molecule and the Cu(100) surface. In this model, the TO[11]H molecule was placed on top of two atomic layers of the Cu(100) surface, with each layer containing 10 Cu atoms arranged periodically along the *a*- and *b*-directions within a simulation cell. The in-plane lattice parameter of the simulation cell is as follows: *a* = *b* = 25.6 Å, which contains a total of 263 atoms. To minimize the artificial interaction between the TO[11]­H/Cu(100) composite and its images along the out-of-plane direction within the periodic setup of the calculation, a vacuum space of ∼28 Å is used. The ab initio study on the model system is performed within the framework of DFT using the all-electron electronic structure code FHI-aims with the ‘light’ default settings for the numerical grid and basis set.^46^ Structural optimization of the model structure is carried out using the exchange-correlation approximated through the PBE formulation of generalized gradient approximation (PBE-GGA).^47^ Dispersive forces were included in the calculations using the Tkatchenko-Scheffler (TS) method,^48^ while relativistic effects were treated on the level of the atomic zero-order regular approximation (atomic ZORA).^46^ The self-consistent field (SCF) cycle was treated as converged when changes of total energy, sum of eigenvalues, and charge density were found below 10^–6^ eV, 10^–3^ eV, and 10^–5^ eÅ^–3^, respectively. During the structural optimization, the bottom Cu layer was constrained, and only the ionic positions of TO[11]H and the top Cu layer were relaxed within the simulation cell until the Hellmann–Feynman forces were reduced below 0.005 eVÅ^–1^. The Brillouin zone was sampled by using the Gamma point. To obtain the PDOS of a reference Cu atom, we performed a PBE0 calculation for the reduced model system after removing the TO[11]H molecule, i.e., for the clean Cu(100) surface.

For stability analysis of the TO[11]­H/Cu(100) interface, the binding energy was evaluated by *E*_binding_ = *E*_model_ – *E*_TO[11]H_ – *E*_Cu(100)_, where *E*_model_ denotes the total energy of the model system containing the TO[11]H molecule located on the Cu(100) surface. *E*_TO[11]H_ and *E*_Cu(100)_ denote the total energy of the TO[11]­H molecule and the Cu(100) surface, respectively. To determine any potential charge transfer between TO[11]H and the Cu(100) surface, the charge density difference is calculated via: Δρ = ρ_model_ – ρ_TO[11]H_ – ρ_Cu(100)_, whereby ρ_model_, ρ_TO[11]H_, and ρ_Cu(100)_ denote the corresponding charge densities of the model, the TO[11]H molecule, and the Cu(100) surface, respectively.

To investigate magnetism at the TO[11]­H/Cu(100) interface, we have employed spin-polarized DFT calculations within PBE as well as the hybrid functional PBE0 schemes,^49^ modified by changing the portion of the Fock exchange (ε_xc_) contributing to this functional. ε_xc_ is varied from 5% to the standard 25 to 35% of nonlocal Fock exchange. To make the calculation tractable within the PBE0 scheme, the system size was reduced from a 10 × 10 to a 6 × 6 supercell. This scaling reduces the number of atoms but does not alter the underlying lattice periodicity in the in-plane directions. Note that the bonding environment around the TO[11]­H/Cu(100) interface remains unaltered by cell reduction. The in-plane lattice parameter of the reduced simulation cell becomes as follows: *a* = *b* = 15.3 Å, containing a total of 135 atoms, thus reducing the number of atoms inside the cell and making it suitable for hybrid calculations. Converged SCF is used to compute the spin density on the reduced model system within both PBE and PBE0 schemes.

#### Model Hamiltonian

To understand the influence of electron–electron interactions and hybridization on the electronic and magnetic properties of the TO[11]­H/Cu(100) interface, a Hubbard model expressed by the following Hamiltonian has been employed:

$$H=H_{\mathrm{HOMO}}+H_{s}+H_{d}+H_{\mathrm{sH}}+H_{\mathrm{dH}}$$ 2

The first term in eq 2 for the adsorbate TO[11]­H molecule, arising from its frontier HOMO orbital, can be expressed as

$$H_{\mathrm{HOMO}}=\sum_{\sigma }(\varepsilon_{\mathrm{HOMO}}C_{\mathrm{HOMO}\sigma }^{†}C_{\mathrm{HOMO}\sigma })+U_{\mathrm{HOMO}}n_{\mathrm{HOMO}↑}n_{\mathrm{HOMO}↓}$$ 3

It consists of two parts: (i) the on-site energy of electrons occupying HOMO and (ii) Coulomb repulsion from double occupancy of HOMO. Here, ε_HOMO_ and *U*_HOMO_ represent the energy level and the on-site Coulomb interaction of HOMO, respectively. The operator *C* ^†^_σ_ (*C*_σ_) creates (annihilates) an electron with a spin σ on any state. *n*_↑_ and *n*_↓_ are the number operators for electrons with spin-up and spin-down, respectively.

The second and third terms in eq 2 represent the Cu(100) substrate. The second term arises from the delocalized 4s states, which are singly occupied and contribute to conduction. The third term originates from the localized 3d states, which are fully occupied and do not participate in conduction. However, they can hybridize with the TO[11]H molecule and, under certain electron correlation, may induce spin polarization, as suggested by the DFT results. For modeling conduction 4s electrons of Cu, we consider a simple tight-binding model of a linear chain of site *i* (*i* = 1,2,...,*m*; *m* = 100) characterized by onsite energy ε_s_ and nearest neighbor hopping integral *T*. Hence, the second term can be expressed as

$$H_{s}=\sum_{i,\sigma }\varepsilon_{s}C_{i,\sigma }^{†}C_{i,\sigma }+T\sum_{ij>,\sigma }(C_{i,\sigma }^{†}C_{j,\sigma }+h.c.)$$ 4

The third term corresponding to the localized 3d electrons can be expressed as

$$H_{d}=\sum_{\sigma }(\varepsilon_{d}C_{d\sigma }^{†}C_{d\sigma })+U_{d}n_{d↑}n_{d↓}$$ 5

Here, ε_d_ and *U*_d_ represent the energy level and on-site Coulomb interaction of d-orbitals, respectively.

The fourth and fifth terms in eq 1 correspond to the coupling of TO[11]H adsorbate on the Cu(100) substrate, representing hybridization of Cu-4s and Cu-3d orbitals with TO[11]­H-HOMO, respectively, and can be expressed as

$$H_{\mathrm{dH}}=t_{\mathrm{dH}}\sum_{\sigma }(C_{d\sigma }^{†}C_{H\sigma }+h.c.)$$ 6

$$H_{\mathrm{sH}}=t_{\mathrm{sH}}\sum_{\sigma }(C_{s\sigma }^{†}C_{H\sigma }+h.c.)$$ 7

Here, *t*_dH_ and *t*_sH_ represent the corresponding hopping integral.

#### Mean Field Approximation

The Hubbard model Hamiltonian was solved by using the Hartree–Fock mean-field approximation under the assumption of collinear spins. Under this approximation, the many-body Coulomb interaction term can be simplified as follows:

$$Un_{i,↑}n_{i,↓}=Un_{i,↑}\left\langle n_{i,↓}\right\rangle +Un_{i,↓}\left\langle n_{i,↑}\right\rangle -U\left\langle n_{i,↑}\right\rangle \left\langle n_{i,↓}\right\rangle$$ 8

where ⟨*n*_*i*,σ_⟩ (σ = ↑, ↓) is the average or “mean-field” spin σ electron density at site *i*. Hence, the problem reduces to an effective single-electron problem characterized by the Mean Field Hubbard (MFH) Hamiltonian, where each electron experiences a mean-field potential that depends on the electron density. The electron density is determined self-consistently from single-electron eigenstates. A random electron density ⟨*n* ^0^_*i*,σ_⟩ was used as a starting guess. Keeping the mean-field electron density constant, the MFH Hamiltonian was divided into spin up and down terms and solved for its single particle eigenvalues *E*_*j*,σ_ and eigenvectors *C*_*i*,*j*,σ_ (*i*: site index, *j*: eigenstate index). Therefore, the electron density can be calculated as

$$\langle n_{i,\sigma }\rangle =\sum_{j}^{N_{\mathrm{el}}}|C_{i,j,\sigma }{|}^{2}$$ 9

The difference between the input and output electron densities was quantified by the residual:

$$Y=\sqrt{\underset{i}{\sum }{\left(\left\langle n_{i,\sigma }\right\rangle -\left\langle n_{i,\sigma }^{0}\right\rangle \right)}^{2}}$$ 10

If the residual was sufficiently small, then the calculation reached self-consistency. Otherwise, the computed ⟨*n*_*i*,σ_⟩ values will be used to update charge densities (⟨*n* ^0^_*i*,σ_⟩) via the linear mixing algorithm:

$$\left\langle n_{i,\sigma }^{0}\right\rangle =\beta \left\langle n_{i,\sigma }^{0}\right\rangle +\left(1-\beta \right)\left\langle n_{i,\sigma }\right\rangle$$ 11

where β is the mixing parameter lying between 0 and 1.

The parameters of interest are the following,

1) The magnetic moments on each site *i*:
$$M_{i}=\left\langle n_{i,↑}^{0}\right\rangle -\left\langle n_{i,↓}^{0}\right\rangle$$ 12
2) The density of states on each site *i* for spin σ:
$${\mathrm{DOS}}_{i,\sigma }(E)=\eta \sum_{j}\frac{|C_{i,j,\sigma }{|}^{2}}{(E-E_{j,\sigma }{)}^{2}+\eta^{2}}$$ 13

where η is the broadening factor.

#### DMRG Calculations

The DMRG method was employed to obtain the ground-state properties of the model Hamiltonian. All DMRG calculations were performed using the ITensor library in Julia.^50^ A truncation error cutoff of 10^–8^ was imposed to ensure high numerical accuracy, while the bond dimension was allowed to grow without an explicit upper bound. The variational optimization of the matrix product state was iterated until the total energy converged within 1 × 10^–8^ eV.

## Supplementary Material



## Notes

LEEM images, work function difference maps, spin asymmetry histogram and image, inverted spin-polarization plot, spin asymmetry plot for (P)-TO[11]­H, charge density difference and spin density plots of adsorbate complex, phase diagrams showing dependence of magnetization on various modeling parameters, and details of HPLC separation (PDF)

## ⊥

Nova Measuring Instruments, Fremont, California 94538, United States
