# Supplementary material for: Adsorption-Induced Surface Magnetism
Source: ACS Nano. 2026 Jan 30;20(5):4143–51. doi: 10.1021/acsnano.5c15791 (PMC12895514; doi:10.1021/acsnano.5c15791)
Supplement: Supplementary file 1 [file nn5c15791_si_001.pdf]

Supporting Information

Adsorption-induced surface magnetism

Miloš Baljozović,[1] Shiladitya Karmakar,[2] André L. Fernandes Cauduro,[3] Mothuku Shyam Sundar,[4] Marco Lozano,[2] Manish Kumar,[2] Diego Soler Polo,[2] Andreas K. Schmid,[3] Ashutosh V. Bedekar,[4] Pavel Jelinek[2]* & Karl-Heinz Ernst[1,2]*

[1] Empa, Swiss Federal Laboratories for Materials Science and Technology; Dübendorf, Switzerland.

[2] Nanosurf Lab, Institute of Physics of the Czech Academy of Sciences; Prague, Czech Republic.

[3] National Center for Electron Microscopy, Molecular Foundry, Lawrence Berkeley National Laboratory; Berkeley, CA 94720, USA

[4] Department of Chemistry, The Maharaja Sayajirao University of Baroda; Vadodara 390 002, India

e-mail: jelinekp@fzu.cz ; kalle@fzu.cz

SI Figures

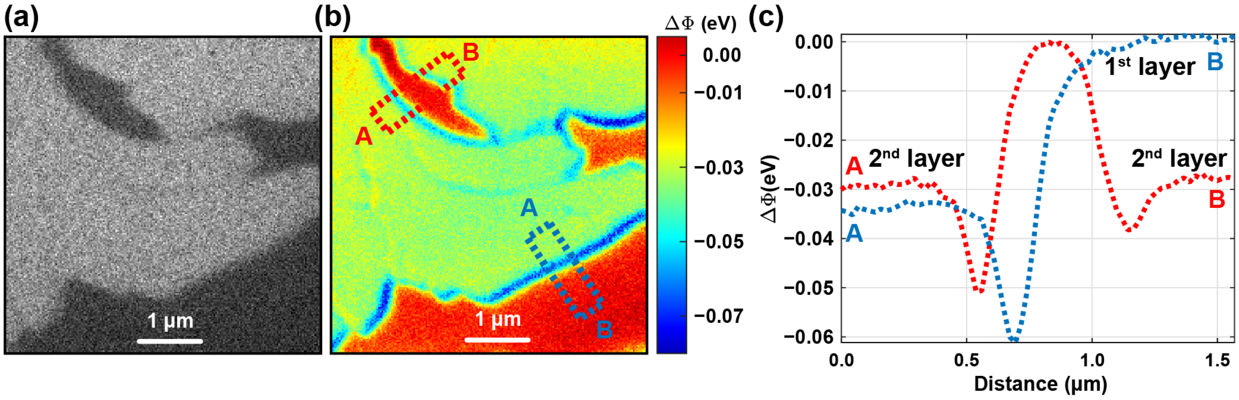

Figure S1. Work function difference between 1st and 2nd layer of *(M)*-TO[11]H. a) LEEM bright field image acquired using start voltage of 3.5 V where first layer appears dark with second layer appearing bright. b) Pixel-by-pixel map of ΔΦ of the film shown in (a) plotted in colour scale shown on the right side of the panel, representing work function difference between the two layers. c) line profiles from areas marked in b) showing that the double layer regions have about 30 meV lower work function with respect to the 1 ML regions. Work-function changes at the boundaries between layers are artifacts originating from the beam drift during the measurements.

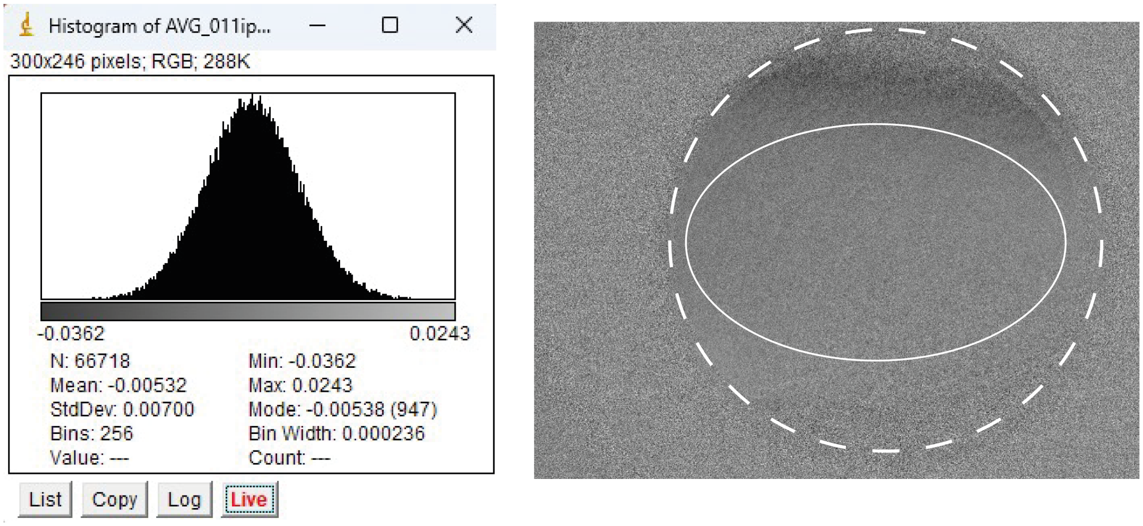

Figure S2. Example spin asymmetry image calculated according to Equation 1 (right). Dashed circle highlights the complete LEEM field of view, while ellipse denotes area in the field of view from which the average spin-asymmetry values were determined. The areas from field of view on top and bottom ends with less homogeneous illumination are discarded. Histogram (left) of spin asymmetry values from the area encircled with the ellipse. The distribution of the values is used for estimating errors of the spin asymmetry values.

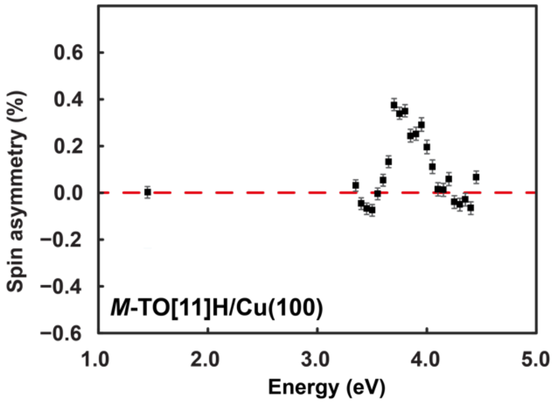

Figure S3. By inversion of the spin-polarization sequence a reversal of asymmetry with respect to the negative dips shown in Figures 2 and S4.

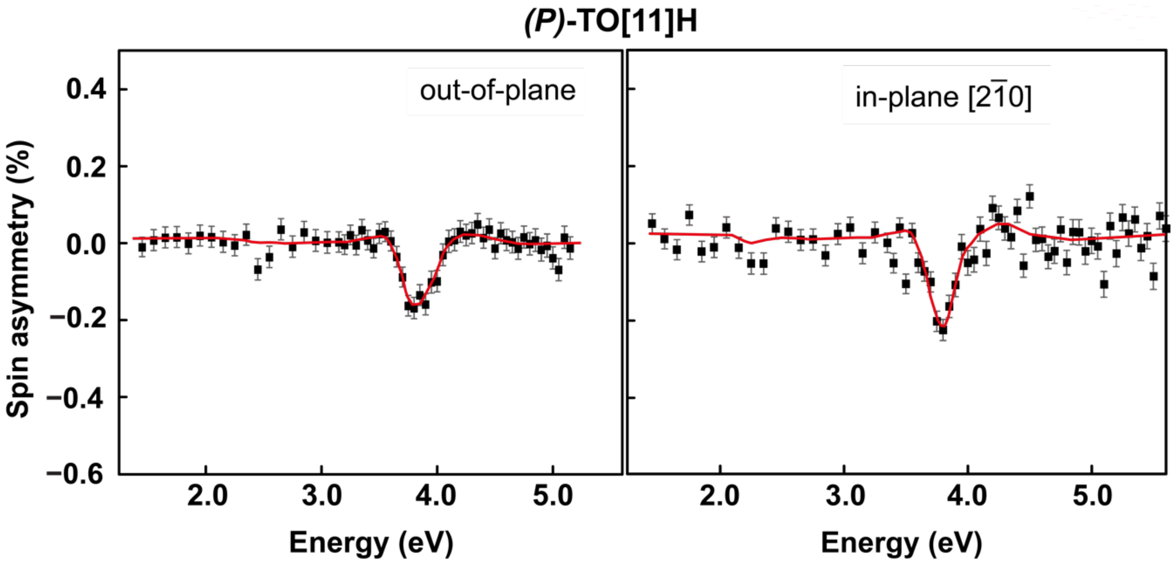

Figure S4. Spin asymmetry plots of *(P)*-TO[11]H on Cu(100). Out-of-plane (left) and in-plane [2$\bar{1}$0] (right) spin asymmetry plots of *(P)*-TO[11]H on Cu(100). No difference compared to the asymmetry plots of *(M)*-TO[11]H on Cu(100) shown in Figure 2 is observed which excludes chirality-induced spin selectivity as origin.

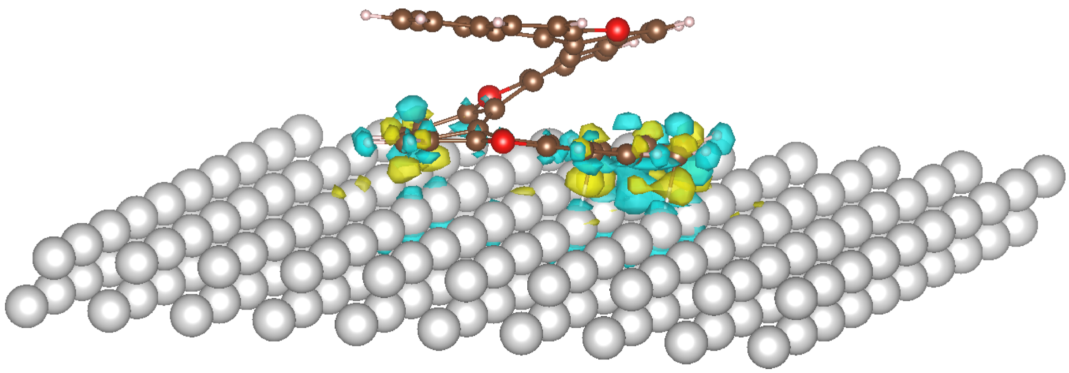

Figure S5. Sketch of the charge density difference Δρ of model system containing the TO[11]H molecule placed on 10×10 Cu(100) slab calculated using the PBE exchange–correlation functional. The isosurface is set at 0.02 eV/Å^3. Charge depletion and accumulation are shown as cyan and yellow isosurfaces, respectively, with $\Delta\rho > 0$ indicating accumulation and $\Delta\rho < 0$ indicating depletion.

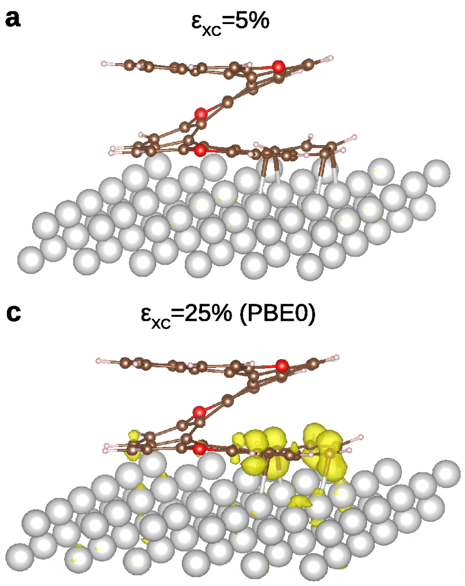

Figure S6. Sketch of the spin density of model system by varying the non-local Hartree Fock exchange (ε_{xc}) that contributes to the hybrid functional PBE0. Spin density accumulation is shown for a) ε_{xc}=5%, b) ε_{xc}=15%, c) ε_{xc}=25%, and d) ε_{xc}=35%. The isosurface is set at 0.02 e/Å^3 with accumulated spin is shown by yellow colored isosurface. Spin density increases as ε_{xc} is increased. ε_{xc} = 25% is the case of standard PBE0.

Phase diagram in the (U_d, t_{dH}) and (U_d, t_{sH}) parameter spaces:

Figure S7 shows phase diagrams of M_{HOMO} in the (U_d, t_{dH}) and (U_d, t_{sH}) planes. Two regions are observed: a non-magnetic phase and a magnetic phase with finite M_{HOMO}. In panel (a), the phase boundary shifts to larger U_d as t_{dH} increases. Suppression of the magnetic solution for large values of t_{dH} is observed. The magnetic phase boundary in the t_{dH}-U_d plane is nearly linear, with slope ~0.3. This indicates that a magnetic solution emerges only when U_d/t_{dH} ~ 3.33. In panel (b), increasing t_{sH} suppresses magnetism, expanding the non-magnetic region. But relatively weak dependence of the U_d threshold on t_{sH} is observed.

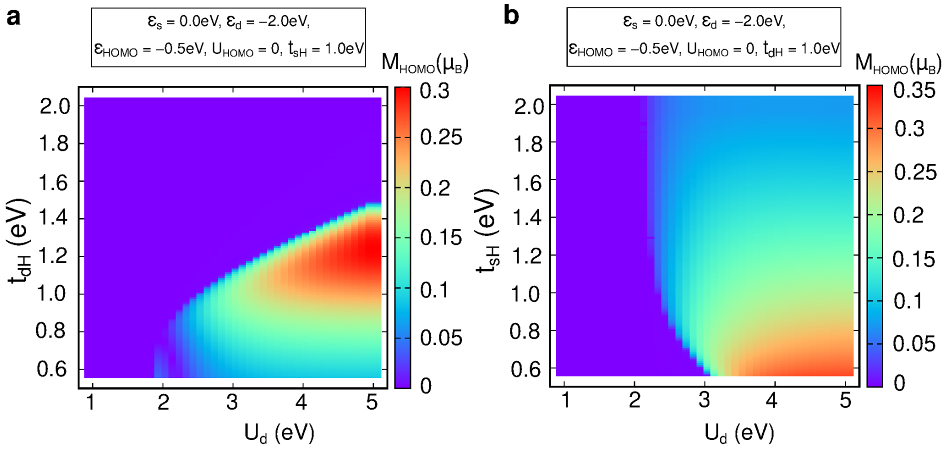

Figure S7. (a) t_{dH}-U_d phase diagram with t_{sH} = 1.0 eV. (b) t_{sH}-U_d phase diagram with t_{dH} = 1.0 eV. In both panels, ε_s = 0 eV, ε_d = −2.0 eV, ε_{HOMO} = −0.5 eV, and U_{HOMO} = 0. Two distinct regions are observed depending of M_{HOMO}: a) non-magnetic region, marked in indigo, b) magnetic region, where $M_{HOMO} < 0.25\mu_B$, marked in green and $M_{HOMO} > 0.25\mu_B$, marked in red.

Phase diagram in the (U_d, U_{HOMO}) and (U_d, ε_{HOMO}) parameter spaces:

Figure S8 shows phase diagrams of M_{HOMO} as a function of U_d and the molecular parameters U_{HOMO} (panel a) and ε_{HOMO} (panel b). Two regions are observed similar to Figure S7. In panel (a), a sharp phase boundary is observed, indicating that the threshold value of U_d is insensitive in U_{HOMO}, thus correlation on the HOMO plays only a minor role. In contrast, panel (b) shows that ε_{HOMO} strongly affects the onset of magnetism: lowering ε_{HOMO} stabilizes spin polarization by reducing the U_d threshold. Notably, magnetism is favored only when the HOMO lies no more than ~ 1.5 eV below E_F. To benchmark the mean-field results, we also calculated the occupations of natural orbitals by solving the model Hamiltonian using DMRG with the same model parameters. From these occupations of natural orbitals, the radical character was evaluated following the method proposed by Yamaguchi et al.,[1] which provides insight into the magnetization of the molecule (Figure S9).

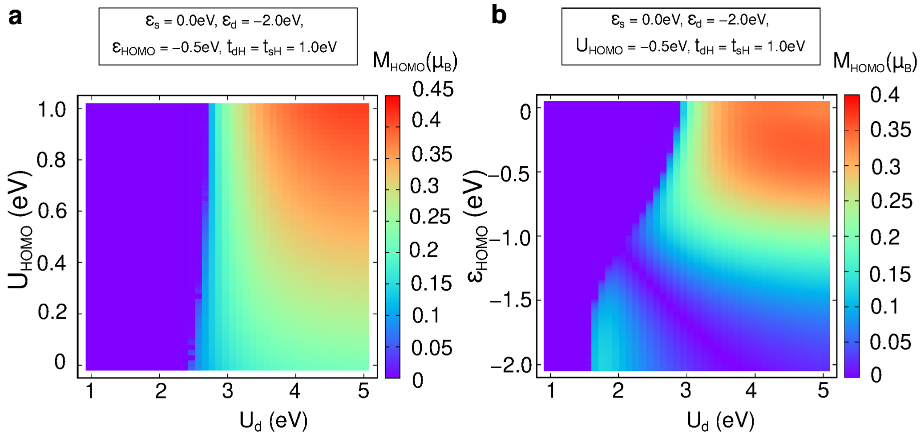

Figure S8. (a) U_{HOMO} vs U_d phase diagram with ε_{HOMO} set at –0.5 eV showing distinct phases depending of M_{HOMO}. (b) ε_{HOMO} *versus* U_d phase diagram with U_{HOMO} set at 0.5 eV. Different regions can be marked as following: i) non-magnetic region (marked in indigo), ii) magnetic region, where $M_{HOMO} < 0.25\mu_B$ (marked in green), and $M_{HOMO} > 0.25\mu_B$ (marked in red).

(1) Yamaguchi, K.; Takahara, Y.; Fueno, T.; Houk, K. N. Extended Hartree-Fock (EHF) theory of chemical reactions. III. Projected Møller-Plesset (PMP) perturbation wavefunctions for transition structures of organic reactions. *Theoret. Chim. Acta* **1988,** *73*, 337–364.

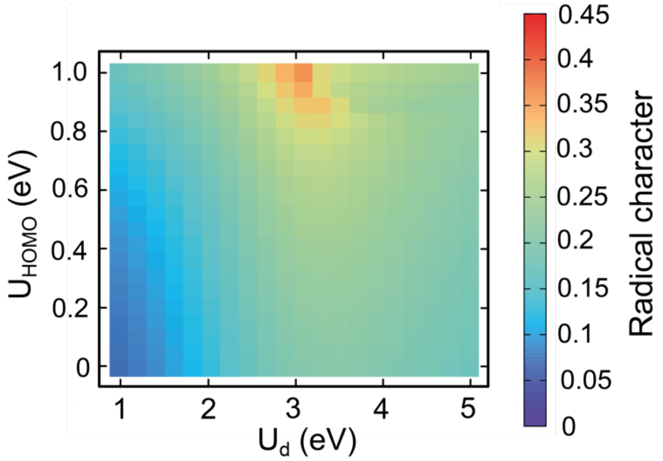

Figure S9. Phase diagram of U_{HOMO} vs U_d, obtained using the same parameters as in Figure S8a, showing the radical character of the molecule. The radical character is calculated using the method proposed by Yamaguchi et al.,[1] based on the occupation number of natural orbitals obtained from the DMRG calculations.

Chiral separation of trioxa[11]helicene on preparative HPLC

The resolution of racemic trioxa[11]helicene into its enantiomers was performed by HPLC using a Chiralart Cellulose SC column (250 x 4.6 mm, 5 um, YMC) and *n*-heptane/2-propanol (95:05) mixture as the mobile phase. The racemic sample is dissolved in *n*-heptane/2-propanol, injected on the chiral column, and detected with an UV detector at 254 nm. The earlier eluting fractions gave the enantiomer exhibiting a positive optical rotation (+) which was isolated in 40% yield and 100% ee. Later eluting fractions consisted of the enantiomer exhibiting a negative optical rotation (-) in 42% yield with > 95% ee. The enantiomeric purity of both enantiomers was checked by chiral HPLC using the same stationary phase. The optical rotations were measured in chloroform using an Autopol IV instrument (Rudolph Research Analytical) and the specific rotation values obtained for (+)-**E1** (*P*-isomer) and (–)-**E2** (*M*-isomer) were found to be $[\alpha]^{25}_{D}$ +3803 (c = 0.160, $CHCl_3$) and –3610 (c = 0.154, $CHCl_3$), respectively. The very high specific rotation value is a known feature of helical structures.

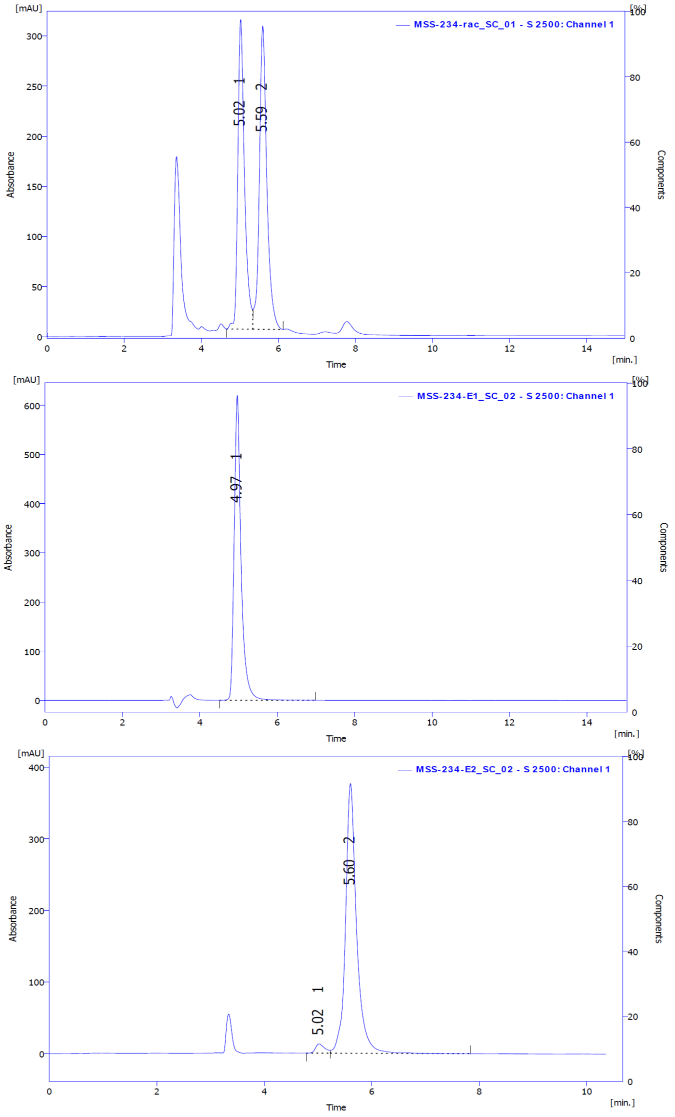

Figure S10. HPL chromatograms of racemic TO[11]H (top) and of the two separated enantiomers (middle, bottom). Column: Cellulose SC (250 x 4.6 mm, 5 um, YMC), Mobile Phase: heptane - i-PrO H 95:5, Flow Rate: 1.0 mL/min., Detection: UV.

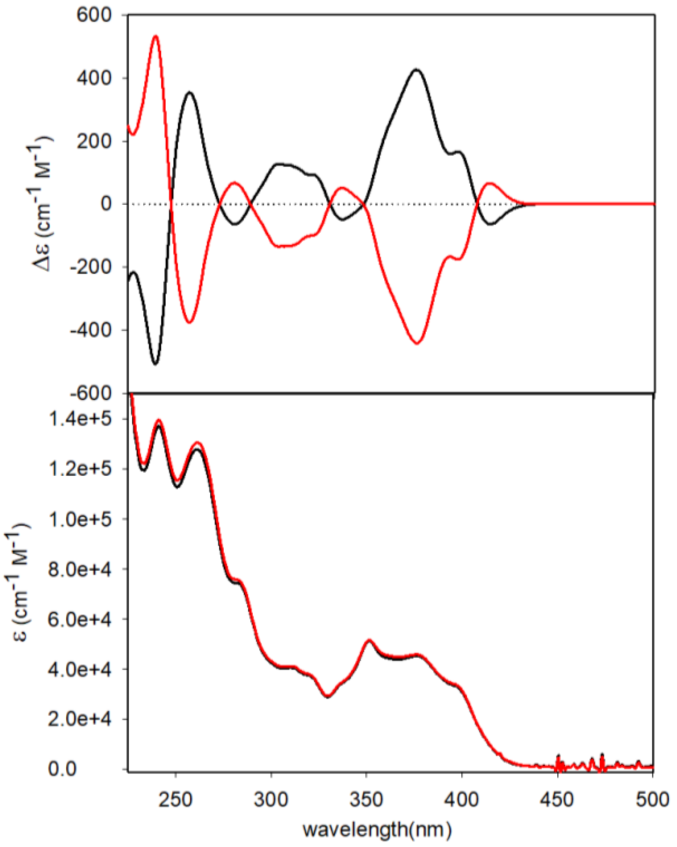

Figure S11. Circular dichroism (upper curve), red line (–)-(*M*)-**E2** and black line (+)-(*P*)-**E1** and UV−vis-absorption (lower curve) of trioxa[11]helicene (concentration = 0.1 mM in THF, 25 °C).

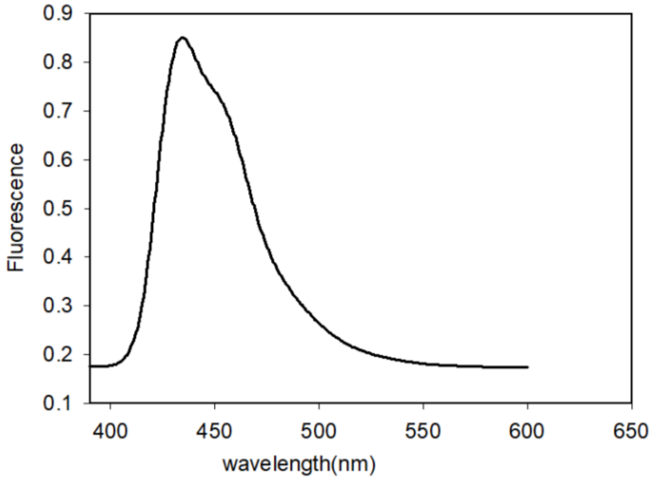

Figure S12. Fluorescence spectrum of trioxa[11]helicene (concentration = 0.1 mM in THF, 25 °C) upon excitation at 395 nm.
